# Exploring the potential of microRNA as a diagnostic tool for gestational diabetes

**DOI:** 10.1186/s12967-023-04269-2

**Published:** 2023-06-17

**Authors:** Duaa Ahmed Elhag, Souhaila Al Khodor

**Affiliations:** Maternal and Child Health Division, Research Branch, Sidra Medicine, Doha, Qatar

**Keywords:** Diabetes, OGTT, Pregnancy complications, BMI, Macrosomia

## Abstract

MicroRNAs (miRNAs) are small non-coding RNAs that play critical roles in regulating host gene expression. Recent studies have indicated a role of miRNAs in the pathogenesis of gestational diabetes mellitus (GDM), a common pregnancy-related disorder characterized by impaired glucose metabolism. Aberrant expression of miRNAs has been observed in the placenta and/or maternal blood of GDM patients, suggesting their potential use as biomarkers for early diagnosis and prognosis. Additionally, several miRNAs have been shown to modulate key signaling pathways involved in glucose homeostasis, insulin sensitivity, and inflammation, providing insights into the pathophysiology of GDM. This review summarizes the current knowledge on the dynamics of miRNA in pregnancy, their role in GDM as well as their potential as diagnostic and therapeutic targets.

## Introduction

According to the World Health Organization (WHO) and the International Federation of Gynecology and Obstetrics (FIGO), Gestational Diabetes Mellitus (GDM) is defined as a pregnancy-related carbohydrate intolerance that is first diagnosed during pregnancy [1, 2]. This results in varying degrees of hyperglycemia and is associated with potential complications such as pre-eclampsia, premature rupture of membranes, cesarean section, preterm delivery, high blood pressure, and babies with large birth weight [3–6]. The worldwide prevalence of GDM is around 14%, varying based on the population ethnicity and the diagnostic test used [6–8]. The American Diabetes Association (ADA) recommends performing the oral glucose tolerance test (OGTT) for the diagnosis of GDM in the second trimester (between 24 and 28 weeks) for low-risk pregnant women, but early diagnosis in the first trimester can identify those at high risk for GDM and prevent adverse complications by adjusting the cut-off points of the OGTT plasma glucose test [9, 10]. Despite that the OGTT can detect up to 80.3% of GDM cases, there is a need for additional diagnostic biomarkers to achieve 100% diagnostic accuracy for GDM cases as early as the first trimester. This would improve outcomes for pregnant women and their infants.

Pregnancy is characterized by physiological and metabolic changes that prepare the mother's body for fetal growth, which is a well-established fact [11, 12]. These include temporal variations in the expression profile of microRNAs (miRNAs), particularly in the first trimester [13]. miRNAs have the potential to identify pregnant women with complications such as preeclampsia (PE), or GDM [13]. These non-coding and highly conserved RNAs are typically 18–22 nucleotides in length and are known to regulate targeted gene expression by binding to their 3'UTR [14]. They are among the most commonly emerging epigenetic regulators for metabolic adaptation during pregnancy [15–17]. However, their dysregulation has been associated with several pregnancy complications, including PE, intrauterine growth restriction (IUGR), miscarriage, preterm birth, and GDM [17–22]. Interestingly, a panel of miRNAs has already been validated for several other diseases, including gastric cancer, breast cancer, and diabetes [23–25], however, there are relatively few studies that have validated the role of miRNAs as a diagnostic biomarker for pregnancy complications, including GDM [26]. Several risk factors, including advanced maternal age, overweight and obesity, macrosomia, history of perinatal complications, diabetes in the family, parity, and Asian ethnicity, are known to play a significant role in the pathogenesis of GDM [6, 27–29]. By correlating these risk factors with other established diagnostic biomarkers, it may be possible to achieve an optimal diagnosis of GDM as early as the first trimester.

The objective of this review is to highlight the most dysregulated miRNAs and their mechanisms of action in pregnant women with GDM, as well as to explore their correlation with other risk factors for GDM.

### Role of microRNA in GDM

#### Up-regulated microRNAs and their mechanism of action

miRNAs have been shown to be involved in the regulation of glucose metabolism and insulin secretion. The expression levels of different miRNAs can vary significantly according to gestational age and across different populations [30–40]. For instance, two previous studies showed a significant overexpression of miR7-5P in maternal blood of women with GDM [41, 42]. In vitro validation of the genetic targets revealed a down regulated expression levels for IRS1/2 (Insulin Receptor Substrate) and RAF genes [30, 31]. This can be mediated by the overexpression of miRNA 7 in women with GDM which highlighted its potential role in regulating insulin, GnRH, and inflammatory signaling pathways associated with IRS1/2 and RAF genes [41, 42]. Interestingly, Mexican women with GDM exhibited significantly upregulated levels of miR-9-5p, miR-29a-3p, and miR-330-3p during the second trimester [30]. Notably, miR-9-5p may contribute to GDM by targeting HK-2 (Hexokinase-2), which in turn regulates genes involved in glycolytic pathways such as GLUT1 (glucose transporter 1), PFK (phosphofructokinase), and LDH (lactate dehydrogenase) [43]. Furthermore, miR-9-5p has also been found to be overexpressed in the serum of newly diagnosed individuals with type 2 diabetes (T2D), suggesting a potential role in glucose metabolism regulation [44].

Additionally, several previous studies have reported elevated levels of miR-16-5p, miR17-5p, miR-19a-3p, miR-19b-3p, and miR-20a-5p in the blood of Chinese and Polish women with GDM as early as 16 weeks, with this correlation increasing and persisting throughout the second and third trimesters [31, 32, 45, 46]. The observed upregulation of miRNAs was also shown to be positively correlated with insulin resistance (IR), a known risk factor for GDM [31]. Network analysis of these upregulated miRNAs has revealed their association with five pathways, namely MAPK signaling, insulin signaling, T2D, TGF-β signaling, and mTOR signaling [46]. Abnormal MAPK signaling is associated with pregnancy complications and sensitivity to insulin, while the TGF-β signaling pathway is linked to PE [47]. Furthermore, the mTOR signaling pathway controls energy balance and food intake in the hypothalamus. [48]. Therefore, the dysregulation of these pathways may play a role in the development of GDM. Remarkably, miR-16 has been demonstrated to target multiple genes that participate in various biological processes such as pancreatic β-cell proliferation and apoptosis [49], insulin signaling [50], and insulin receptor substrate (IRS) proteins 1 and 2 which are involved in insulin-like growth factor-I (IGF-I) regulation, a factor closely linked to insulin resistance [46, 51–53]. MiR-16 has been found to impact insulin resistance and inhibit cell apoptosis induced by hyperglycemia, by targeting genes involved in biological processes such as insulin signaling, insulin receptor substrate (IRS) proteins 1 and 2, and insulin-like growth factor-I (IGF-I) [50]. Although miR-17-5p and miR-16-5p have been associated with T2D and other metabolic diseases, their exact role in the pathogenesis of GDM remains unclear [54]. Notably, an increase in miR-16-5p expression has been observed during hypoglycemic episodes in individuals with T1D or T2D, with a negative correlation observed with interleukin (IL)-6, intercellular adhesion molecule (ICAM), and vascular cell adhesion molecule (VCAM) [55] (NCT03460899). Moreover, miR-16 has been found to exert distinct anti-inflammatory effects by promoting the secretion of anti-inflammatory factors such as IL-10 and TGF-β, while simultaneously reducing the levels of pro-inflammatory factors including IL-6, TNF-α, MCP-1, and IL-1β [56]. These complex anti-inflammatory mechanisms are mediated through downregulation of several targets including nuclear factor-κB (NF-κB) or NOD-like receptor protein 3 (NLRP3) inflammasome [57]. MiR-16 is a down-regulated target of Toll-like receptor 4 (TLR4) and has been found to be upregulated in patients with acute myocardial infarction [57]. For microRNA-20a-5p, one study has shown its upregulation in pre-eclampsia, which has a close relationship with GDM [58], however, its correlation with IR needs further clarification. In a recent study of 82 European obese pregnant women, a distinct dysregulated pattern of miR-16-5p, -29a-3p, 103-3p, 134-5p, -122-5p, -223-3p, -330-3p, and miR-433-3p was observed throughout pregnancy in both GDM and control groups, nonetheless, the initial increase of miR-433-3p was significant only in the GDM group [33] as shown in Table 1. Furthermore, miR-195-5p has been shown to target genes involved in fatty acid metabolism, particularly during the second trimester [37].

**Table 1:** Previous studies on dynamics of the most common significantly dysregulated miRNA in pregnant women with GDM

Population ethnicity	GDM diagnostic criteria	Gestational age at miRNA detection	Sample size	Type of sample	miRNA detection method	Normalization To correct for technical variation	Significantly Dysregulated MiRNA in GDM group and the study findings	Refs.
Mexican (Hispanic/Latino ethnicity is a predisposing factor)	International Association of Diabetes Pregnancy Study Group (IADPSG)	16–19 weeks	40 (GDM = 18, control = 22)	Serum	Real-Time PCR System	miR-454	-Higher expression of miR-9-5p, miR-29a-3p and miR-330-3p No significant difference of miR-16-5p expression	[30]
Chinese	NA	24–28 weeks	(GDM = 85, control = 72)	Plasma	Real-Time PCR System	*Caenorhabditis elegans* (C. elegans) microRNAs (cel-miR-39, cel-miR-54, and cel-miR-238)	microRNA-16-5p, -17-5p and 20a-5p were significantly upregulated	[31]
Chinese	American Diabetes Association (ADA)	16-19 weeks	(GDM = 10, control = 10)	Plasma	high-throughput sequencing technology	Digital Gene Expression II and validated by qRT-PCR	miR-16-5p, miR17-5p, miR-19a-3p, miR-19b-3p and miR-20a-5p were significantly upregulated	[46]
Poland	World Health Organization (WHO) criteria	9–12 weeks	(GDM = 24, control = 24)	Serum	NanoString technology	average geometric mean of the top 100 probes detected	microRNA-16-5p, miR-142-3p and miR-144-3p were significantly upregulated	[32]
European	IADPSG/WHO2013 criteria	baseline ≤ 19 ± 6 days 24–28 weeks 35–37 weeks	obese women with GDM (n = 41), control (n = 41)	Serum	ViiA real-time PCR System	Caenorhabditis elegans (cel)-miR-39, ath-miR-159) and the endogenous small nuclear U6	Initially significant increase of miR-433-3p while levels of miR-122-5p, -223-3p and -16-5p were significantly higher in the GDM group by the third trimester	[33]
Chinese	NA	24–28 weeks	GDM (n = 100) Control (n = 100)	Serum	Quantitative real-time PCR (qPCR)	the endogenous small nuclear U6	4.0 fold increase in miRNA-19a and 4.7 mean increase in miRNA-19b expression	[45]
Caucasian	International Association of the Diabetes and Pregnancy Study Groups (IADPSG)	24–32 weeks	Screening group: GDM (n = 8) Control (n = 8) Validation group: : GDM (n = 30) Control (n = 30)	WBCs Cord blood	q-PCR	the house keeping gene U6 small nuclear 6	Significant increase of miRNA-340 marginal increase of 142, miRNA-143 and let-7 g	[34]
American	American Diabetes Association (ADA) 2004 criteria	7–23 weeks	GDM (n = 36) controls (n = 80)	Plasma	qRT-PCR	an endogenous housekeeping miRNA, miR-423-3p	miR-155-5p and—21-3p levels were signigicantly increased miR-146b-5p and miR-517-5p were borderline. Associations of miR-21-3p and miR-210-3p with GDM were observed among overweight/obese but not lean women. Associations of six miRNAs (miR-155-5p, -21-3p, -146b-5p, -223-3p, -517-5p, and -29a-3p) with GDM were present only among women carrying male fetuses	[35]
Canadian	guidelines of the Society of Obstetricians and Gynecologists of Canada	6–15 weeks	GDM (n = 23) controls (n = 46)	extracellular vesicles in serum	Quantitative real-time PCR	the spike-in control synthetic *Caenorhabditis elegans* miR-39-5p	miR‒122-5p; miR‒132-3p; miR‒1323; miR‒136-5p; miR‒182-3p; miR‒210-3p; miR‒29a-3p; miR‒29b-3p; miR‒342-3p, and miR-520 showed significantly higher levels	[36]
European	IADPSG/WHO2013 criteria	15.1 ± 2.4 weeks	GDM (n = 82) controls (n = 41) from obese pregnant women	Serum	qPCR assays	Synthetic *Caenorhabditis elegans* (cel)-miR-39 was	Elevated miR-16-5p, -29a-3p, and -134-5p levels in women, who were NGT at baseline and later developed GDM	[33]
Estonian	International Association of Diabetes in Pregnancy Group’s Consensus Panel criteria	23–31 weeks	GDM (n = 13) Control (n = 9)	plasma	real-time PCR	synthetic C. elegans miR-39	Significant upregulation of let-7e-5p, let-7 g-5p, miR-100-5p, miR-101-3p, miR-146a-5p, miR-18a-5p, miR-195-5p, miR-222-3p, miR-23b-3p, miR-30b-5p, miR-30c-5p, miR-30d-5p, miR-342-3p, miR-423-5p, miR-92a-3p	[37]
Chinese European (Indian–Pakistani) (Latin–American)Filipinos	ADIPS and WHO recommendations	< 18 weeks 22–28 weeks 37–40 weeks	Discovery cohort: cases = 15 controls = 14 Validation cohort: cases = 8, control = 14	Plasma	miRNA sequencing and qRT PCR	housekeeping gene RNU6B	let-7i-5p, miR-10a-5p, miR-151b, miR-16–2-3p, miR-16-5p, miR-1910-5p, -miR-423-5p, miR-92a-3p, miR-92b-3p, miR-563 were significantly upregulated	[68]
Mexican	American Diabetes Association [ADA]	1st trimester 2nd trimester 3rd trimester	GDM (n = 67) Control (n = 74)	Serum	q-RT PCR sequencing	synthetic Cel-miR-39-3p	1^st^ trimester: higher levels of miR-183-5p, -200b-3p 2^nd^ trimester: higher level of miR 125-5p 3^rd^ trimester: higher levels of miR-137	[72]
Mexican	WHO criteria	1st trimester 2nd trimester 3rd trimester	GDM (n = 27) Control (n = 34)	Urine	q-RT PCR	U6 snRNA	significantly higher levels of miR-16-5p, miR-222-3p, miR-516-5p, miR-517-3p and miR-518-5p only in 2nd but not in 3^rd^ trimester	[85]
Italian	Italian guidelines	24–33 weeks	Discovery cohort: GDM (n = 4) Control (n = 4) Validation cohort: GDM (n = 21) Control (n = 10)	Plasma	TaqMan miRNA Human Array Panel A platform qRT real-time PCR	the spike-in control ath-miR-159a	miR-330-3p and miR-483-5p were upregulated, miR-548c-3p and miR-532-3p were downregulated	[38]
Mixed (Spanish and Irish)	the criteria of the National Diabetes Group (NDDG)	third trimester	GDM (n = 31) Control (n = 29)	Serum	qRT real-time PCR	Synthetic C. elegans miRNA (cel-miR-39) spike-in control was added (50 pmol) to each sample for input normalization prior to RNA isolation	miR-330-3p was significantly upregulated which was associated with better response to treatment (diet vs. insulin)	[39]
Chinese	NA	NA	GDM (n = 48) Control (n = 46)	placenta‐derived mononuclear macrophages	qRT‐PCR	NA	significant increased level of miR‐657 which was correlated with reduced revel of IL‐37	[92]
Chinese	the Endocrine Society criteria	after delivery	GDM (n = 15) Control (n = 15)	Placental tissue	miRNA microrarray and real-time PCR (qRT-PCR)		miR-508-3p was up-regulated and miR-27a, miR-9, miR-137, miR-92a, miR-33a, miR-30d, miR-362-5p and miR-502-5p were down-regulated	[95]
Chinese	ADA guidelines	16–19 weeks	-Discovery cohort: GDM (n = 24) Control(n = 24) -Internal Validation cohort: GDM (n = 36) Control(n = 36) -External Validation cohort: GDM (n = 16) Control(n = 16)	Serum	TLDA chip assays and real-time PCR (qRT-PCR)	synthetic C.elegans miR-39	-miR-132, miR-29a and miR-222 were significantly downregulated - knockdown of miR-29a could increase Insulin induced gene 1 (Insig1) expression level which in turn increase the level of Phosphoenol pyruvate Carboxy Kinase2 (PCK2) in HepG2 cell lines	[97]
Chinese	NA	beyond 37 weeks	GDM (n = 204) Control(n = 202)	Placenta	qRT-PCR	U6 snRNA	-miR-29b expression was downregulated targeting HIF3A -miR-29b knockdown promoted trophoblast cell migration	[98]
Chinese	NA	beyond 37 weeks	GDM (n = 166) control(n = 196)	Placenta	tissue microarray in situ hybridization qRT-PCR	U6 snRNA	-miR-30d-5p was significantly down-regulated in GDM placental tissue -in vitro downregulation enhances glucose uptake and regulates HTR8 cells migration and invasion via targeting RAB8A gene	[99]
South African	International Association of Diabetes and Pregnancy Study Group (IADPSG)	13–31 weeks	81 GDM (n = 28) Control (n = 53)	Serum	quantitative real-time PCR	Caenorhabiditis elegans miR-39	miRNA 20- 5p and miR-222-3p were significantly decreased	[40]
Chinese	American Diabetes Association	Post delivery	246 GDM (n = 123) Control (n = 123)	Placenta	quantitative real-time PCR	U6 the housekeeping gene	a significantly lower expression level of miR-96 showing a high sensitivity and specificity	[111]
Turkish	International Association of Diabetes and Pregnancy Study Groups criteria	32–33 weeks plus	69 PCOS (*n* = 17) GDM (*n* = 14) PCOS + GDM (*n* = 11) control (*n* = 27)	Blood	quantitative real-time PCR	RNU6	-a significantly upregulated miR-16-5p expression in PCOS patients -a significantly lower expression level of miR-155-5p in GDM patients showing a positive association with BMI and blood glucose levels	[96]
Chinese	American Diabetes Association (ADA) guidelines	third trimester	GDM (n = 11) control (n = 12)	Plasma	qRT-PCR	NA	significant increase in the expression level of miR-137 that showed to enhance the inflammatory reaction in GDM	[94]
Chinese	NA	NA	GDM (n = 20) Control(n = 20)	peripheral blood	qRT-PCR	U6	Significantly lower level miR-494 showing a novel miR-494/PTEN signaling cascade in GDM	[101]

^*^PCOS polycystic ovary syndrome

MiR-122-5p was significantly upregulated solely in the third trimester [33]. Interestingly, a previous study conducted on individuals with T2D revealed a negative correlation between miR-122-5p and *Bacteriodes uniformis and Phascolarctobacterium Faecium* [59]. Considering the metabolic adaptation that occurs during pregnancy is similar to that in metabolic syndrome, it is plausible to suggest a potential association between miRNA and gut microbiota in the regulation of key genes involved in glucose metabolism [59, 60]. Despite showing a positive correlation with gestational weight gain, miR-433-3p has been found to maintain pancreatic beta cell function in high-glucose conditions, indicating a potential role in protecting against diabetes [33, 61].

In normal pregnancies, the induction of endothelial cell apoptosis by trophoblast cells is a crucial mechanism for uterine spiral artery remodeling [62]. However, defective remodeling has been linked to pregnancy complications such as PE and IUGR [63, 64]. MiR-17-5p expression was found to be significantly higher in 30 Turkish women with GDM, with regulatory effects on mitochondrial fusion-related proteins (Mfn1/Mfn2) in trophoblast cells, affecting endothelial cell apoptosis [65]. This upregulation was positively correlated with fasting glucose levels, HbA1C, and total cholesterol, which are known to be associated with endothelial and vascular dysfunction [65]. Since diabetes is known to be associated with endothelial and vascular dysfunction [66], it is becoming a promising biomarker of GDM. MiR-19a and miR-19b were found to have a higher expression level in the blood of Chinese pregnant women with GDM, primarily during the second and third trimesters, though further validation in a larger group is necessary [45]. These miRNAs were also associated with GDM risk factors, such as age, alcoholism, and smoking, which could potentially exacerbate the disease [45]. Moreover, three studies, as detailed in Table 1, have demonstrated a positive association between miR-29a and GDM in women from Canada, Mexico, and various regions in Europe [30, 36, 67]. In addition, miR-155-5p and miR-21-3p were found to have significantly higher plasma expression levels in overweight and obese American women with GDM [35]. These findings suggest that obesity and fetal gender may play a role in the changes in miRNAs observed in women with GDM. However, they need to be confirmed in larger cohorts comprising diverse ethnic and socioeconomic backgrounds and a wider selection of candidate miRNAs.

Multiple miRNAs were also overexpressed in GDM women from different ethnic groups including miR-16–2-3p, miR-1910-5p and miR-92a-3 (Table 1) [37, 68]. These miRNAs showed a positive correlation with the increased pre-pregnancy BMI [69] which can be mediated by modulating the metabolic activity since the higher concentration of the circulatory miR-92a-3p is inversely linked to the metabolic activity of the brown adipose tissue [70] indicating an impaired metabolic status and increased insulin sensitivity [71]. Furthermore, transfection of skeletal muscles with miR-92a-3p appears to affect the expression of genes involved in Janus kinase/signal transducers and activators of transcription (JAK/STAT) signaling pathways, as well as those associated with T2D and hyperglycemia pathways, underscoring its ability to regulate glucose metabolism in response to insulin within skeletal muscle cells [68]. Interestingly, miRNAs implicated in neural development, including miR-183-5p and miR-200b-3p, exhibit increased expression levels during the first trimester in Mexican women with GDM compared to controls, which may be linked to alterations in neurogenesis and cell proliferation (as delineated in Table 1) [72]. Although several studies have reported elevated expression levels of circulatory miR-142, miR-144-3p, and miR-143 in Chinese, Turkish, and German women with GDM compared to controls [31, 37, 74], these results have yet to be validated in larger cohorts [32, 34, 42]. Notably, both miR-144-3p and miR-142 have been shown to be upregulated in peripheral blood mononuclear cells of individuals with T1D and T2D, as well as in women with GDM [73] indicating an overlapping effects in all types of diabetes.

Overexpression of miR-142-3p in the blood and embryonic tissue of GDM-induced mice showed to promote the proliferation β-cells through targeting FOXO1 gene which is known to control glycogenolysis and gluconeogenesis [74, 75]. Overexpression of miR-142-3p was observed only in pre-T2D women, showing a positive association with insulin, HOMA-IR, BMI, adiponectin, and leptin levels in in obese individuals [76–78]. In addition, upregulated expression and a positive correlation with HOMA-IR of circulating miR-144-3p were observed in a Chinese cohort with impaired fasting glucose making it a predictor of T2D development [79]. Higher circulatory levels of let-7 g was observed in Estonian and Caucasian women with GDM [34, 37]. However, in the Caucasian population, this higher expression was solely reported in the screening group and not in the validation group [34]. The regulatory function of the miRNA-let-7 family in the glucose metabolism is widely recognized, with altered expression levels being associated with metabolic disorders such as T2D [80, 81] indicating similarities in different miRNA induced metabolic pathways. Two studies investigated higher expression of miR-195 in plasma of Estonian and Chinese women with GDM compared to controls [37, 82] which was positively associated with increased BMI, obesity and fasting blood glucose level in patients with metabolic syndrome [83], indicating that aberrant expression of miR-195-5p might function as a novel diagnostic biomarker for GDM.

Australian women with GDM showed elevated levels of miR-197 in their placental exosomes, which were found to be correlated with insulin sensitivity in skeletal muscle tissues [84]. In contrast, Mexican women with GDM had higher expression levels of miR-16-5p and miR-222 only during their second trimester, as detected in placental exosomes isolated from urine samples. [85]. Most of genes that are targeted by miR-16-5p and miR-222-3p are involved in the insulin resistance pathway [54, 86] highlighting the role of these miRNAs in modulating different metabolic processes in women with GDM. Moreover, multiple miRNAs including miR‒122-5p; miR‒210-3p; miR‒29a-3p; miR‒29b-3p; miR‒342-3p, and miR-520 h (Table 1) showed significantly higher levels in GDM cases than in controls. These miRNAs are involved in trophoblast proliferation/differentiation as well as in insulin secretion/regulation and glucose transport during pregnancy [36]. In addition, two independent studies found elevated levels of miR222 in omental adipose tissue and plasma samples collected from Chinese and Canadian women with GDM, respectively [37, 87]. Interestingly, miR222 has been shown to impact glucose uptake in mature adipocytes by regulating the expressions of estrogen receptor ERα and insulin-sensitive membrane transporter GLUT4, suggesting its potential as both a biomarker and therapeutic target for GDM [87]. Additionally, two other studies reported increased levels of miR-223 in serum and plasma samples from women diagnosed with GDM, originating from Italy, Spain and Egypt, respectively [88]. This was correlated with the increased levels of angiopoietin-like protein 8 (ANGPTL8) in addition to lipid markers and fasting blood glucose [88]. Upregulation of miR-330 was observed in serum and plasma of Italian, Mexican, Spanish, and Turkish women with GDM compared to controls as shown in Table 1 [30, 38, 39]. MiR-330-3p is known to target genes involved in beta-cell proliferation and differentiation in addition to insulin secretion, such as E2F1, CDC42 and AGT2R2 [38].

**Fig. 1:**
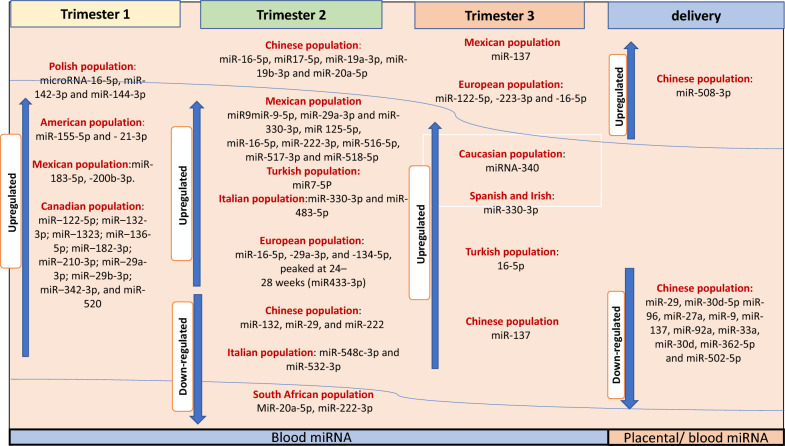
Dynamics of the most common significantly dysregulated miRNA in pregnant women with GDM. List of upregulated and downregulated miRNAs during pregnancy progression.

**Fig. 2:**
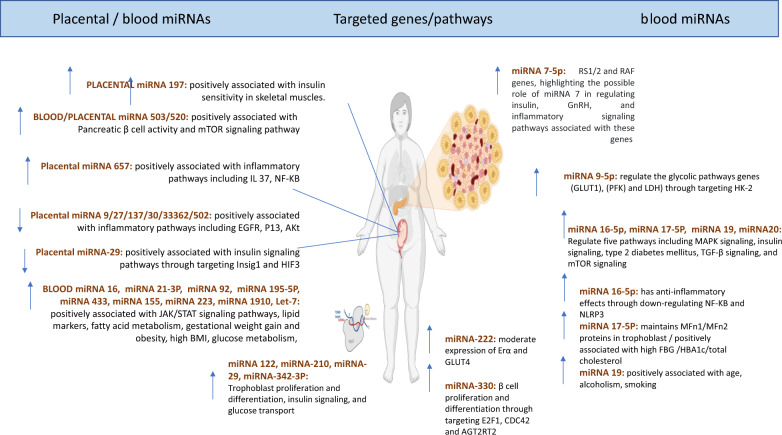
Mechanisms of action for the most common significantly dysregulated miRNA in pregnant women with GDM

In a previous study, miRNA 340 was significantly elevated in GDM patients [34]. While not all GDM subjects exhibited this elevation in comparison to their matched control group, it was positively linked with insulin levels and BMI, as well as the expression levels of the Poly (A) Binding Protein Interacting Protein 1(PAIP1) gene in these women [34]. Interestingly, miRNA-340 has been recently identified as being differentially expressed in diabetic conditions, such as newly diagnosed T1D children [89]. Functional investigations have shown that miRNA-340 responds to insulin and glucose stimuli in cultured lymphocytes suggesting that it may play a crucial role in the alterations in gene expression induced by hyperinsulinemia [34]. Furthermore, miRNA 503 was found to be upregulated in both blood and placenta samples obtained from women with GDM [90]. Notably, miR-503 has been shown to regulate pancreatic β-cell activity by targeting the mTOR pathway, implying that targeting the miR-503/mTOR axis could be a promising therapeutic strategy for GDM [90]. Interestingly, miRNAs isolated from extracellular vesicles in blood, such as miR-520 h, miR-1323, miR-136-5p, and miR-342-3p, were also significantly upregulated in women with GDM [36, 91] Among these, miR-520 h was found to inhibit cell viability and promote cell apoptosis by regulating mTOR expression in a GDM cell model [91]. Additionally, miR-1323 was shown to suppress trophoblast cell viability by downregulating the expression of TP53INP1 gene, highlighting its potential as a therapeutic target for GDM. Moreover, two separate studies reported higher expression levels of miR-657 in placental and placental-derived mononuclear macrophages in women with GDM [92, 93]. The dysregulation of miR-657 has been shown to impact the placental inflammatory response in GDM through its targeting of the IL-37/NF-κB signaling axis [92, 93]. Additionally, it regulates macrophage proliferation, migration, and polarization by targeting FAM46C, suggesting that it holds promise as both a diagnostic and therapeutic target for GDM [92, 93]. While many other miRNAs have been investigated in various studies, we have specifically focused on the most common and significant miRNAs (as depicted in Figs. 1 and 2), along with their known mechanisms of action, highlighting their potential clinical applications for GDM patients.

#### Controversial and down-regulated miRNA profiles in GDM

In the preceding section, we provided a summary of the most prevalent and significant upregulated miRNAs in women with GDM. In this section, we have shifted our focus to the controversial and significantly down-regulated miRNAs. While several miRNAs were found to be significantly upregulated across various GDM cohorts, there were also conflicting results. For instance, miR-9-5p and miR-137 demonstrated a significant upregulation pattern in the blood of women with GDM (as illustrated in Table 1). However, in placental tissues from women with GDM and an associated risk of macrosomia, the opposite—a down-regulated level—was observed [30, 94, 95].

Furthermore, miRNAs such as miR-27a, miR-137, miR-92a, miR-33a, miR-30d, miR-362-5p, and miR-502-5p were downregulated in women with GDM, and they are known to target the epidermal growth factor receptor/Class I phosphoinositide—3 kinases (EGFR/PI3K/Akt) pathway, which sheds light on the potential mechanisms underlying GDM and the associated risk of macrosomia [95]. Although some miRNAs, such as miR-16-5p, miR-17-5p, and miRNA 19, were significantly increased in Chinese and Turkish women with GDM and polycystic fibrosis [45, 96], their association with GDM was not observed in Caucasian and South African women [34, 40]. As previously mentioned, the expression levels of certain miRNAs in women with GDM show variations among different ethnic groups. For instance, miR-20a-5p was found to be significantly upregulated in Chinese pregnant women with GDM, while a study on South African pregnant women reported a significant downregulation of miR-20a-5p and miR-222-3p in the GDM group, which affected genes not related to GDM [31, 40, 46]. Meanwhile, American women with GDM showed no significant difference in the expression of miR-222 and miR-223 in their plasma [35]. Interestingly, miR-29a was found to have a significantly higher level in plasma of Canadian women with GDM, but two previous studies on Chinese women with GDM showed a significantly downregulated pattern of miR-29a and miR-29b in serum and placental samples, respectively [35, 36, 97, 98].

These down-regulated miRNAs can partially play a role in the pathogenies of GDM through modulating glucose metabolism and Placental trophoblast development via targeting the Insulin-induced gene 1 (Insig1) and Hypoxia Inducible Factor 3 Subunit Alpha (HIF3A) gene by miRNA 29a and 29b respectively [97, 98]. Furthermore, miR-30d expression in the placenta has been found to be significantly associated with GDM, as it enhances trophoblast proliferation and glucose uptake capacity by targeting Ras-Related Protein Rab-8A (RAB8A) gene [99]. On the other hand, a lower expression of miR-96 in placental and blood samples of women with GDM has been reported, which affects trophoblast viability and promotes the functions of pancreatic β cells via targeting P21 Protein (Cdc42/Rac)-Activated Kinase (PAK1) gene [105, 106]. These findings shed light on the mechanisms and diagnostic targets of GDM. However, there are conflicting results regarding miR-21 and miR-155, which have been found to be significantly elevated in plasma of American women with GDM [35], but not in Turkish women [96, 100].

The lower expression of miR-21 and miR-155 in Turkish women has been linked to both GDM and PE, suggesting that the expression pattern of miRNA can be influenced by the presence of other pregnancy complications [100]. In Chinese women with GDM, a significant downregulation of miR494 in peripheral blood samples was observed, which inhibited pancreatic β-cell function by targeting the Protein Tyrosine Phosphatase (PTEN) signaling cascade, highlighting a potential therapeutic target for GDM [101]. However, a trend towards increased levels of miR-494-3p was observed in the serum of Canadian women with GDM, but this was not statistically significant [36]. In Italian women with GDM, a downregulation of both miR-548c-3p and miR-532-3p was observed in the screening group but not the validation group [38]. These findings suggest that variation in the miRNA profiles can arise from differences in sample type, gestational age, ethnic group, and the presence of other pregnancy complications. To develop a novel diagnostic panel for GDM, it is useful to focus on the most common dysregulated miRNA profiles across populations (as shown in Figs. 1 and 2) and to replicate these findings in larger pregnancy cohorts.

#### Future directions for use of miRNA as a diagnostic tool

This review has highlighted the significant variability in miRNA expression across different studies, which can be influenced by various factors including medication use, diet, physical activity, ethnicity, socioeconomic and environmental factors, and viral infections [102–106]. Another factor is the variations in the gestational ages between pregnant women [85]. Furthermore, technical factors such as sample collection and storage, miRNA isolation procedures, measurement platforms, and normalization methods can also affect miRNA expression levels [107–109]. To improve reproducibility across studies, standard protocols for sample collection, transport, and storage, as well as miRNA isolation procedures and data analysis, should be developed. Using miRNA panels rather than individual miRNAs can also enhance their clinical applicability, given their ability to regulate multiple genes involved in different biological processes in various diseases [104, 110].

Technological advancements in sequencing can pave the way for the use of miRNAs as inexpensive clinically applicable biomarkers in the future, although pre-analytical, analytical, and biological challenges must first be addressed to overcome poor reproducibility between studies. Although OGTT remains the gold standard diagnostic test for GDM, it is primarily applicable between 24 and 28 weeks of gestation. Therefore, early diagnosis may only be possible by lowering the glucose level cut-off points. Thus, additional early diagnostic markers such as miRNAs are necessary to achieve optimal diagnosis of GDM as early as the first trimester, enabling timely treatment to prevent potential complications of GDM.

In conclusion, dysregulated miRNAs in women with GDM have the potential to serve as noninvasive biomarkers, aiding in the identification of underlying mechanisms for gestational diabetes and associated pregnancy complications. Advanced functional studies are necessary to validate and improve our understanding of these miRNAs by investigating their target genes and pathways. Such studies may help to uncover the link between GDM subtypes and pregnancy outcomes, providing valuable insights into the pathogenesis of GDM.

## Data Availability

Not applicable.
